# A Review of Metal Oxide-Based Materials for U(VI) Sequestration from Aqueous Media

**DOI:** 10.3390/nano16201332

**Published:** 2026-10-21

**Authors:** Weiqiang Sun, Tingting Zhou, Jing Tang, Yitong Zhou, Yiheng Guo, Junqi Wang

**Affiliations:** 1 School of Nuclear Science and Technology, Xi’an Jiaotong University, Xi’an 710049, China; sunweiqiang@xjtu.edu.cn; 2 School of Human Settlements and Civil Engineering, Xi’an Jiaotong University, Xi’an 710049, China; 372498563@stu.xjtu.edu.cn (T.Z.); j.tang@stu.xjtu.edu.cn (J.T.); zhouyitong@stu.xjtu.edu.cn (Y.Z.); g2436390653@stu.xjtu.edu.cn (Y.G.)

**Keywords:** U(VI) sequestration, metal oxides, surface chemistry, uranium retention mechanisms, uranium recovery

## Abstract

Uranium contamination in aquatic environments has raised increasing concerns because of its chemical toxicity and radioactivity, creating a persistent demand for efficient uranium sequestration and recovery technologies. Metal oxides have attracted considerable attention as materials for U(VI) sequestration owing to their chemical stability, tunable structures, and abundant reactive surface sites. This review systematically summarizes the fundamental chemistry and recent development of metal oxide-based materials for U(VI) sequestration from aqueous environments. The aqueous speciation of U(VI), surface chemistry of metal oxides, and major interfacial interactions governing uranium retention are first discussed. Representative materials, including iron oxides (FeOx), titanium oxides (TiO2), aluminum oxides (Al2O3), manganese oxides (MnOx), zirconium oxide (ZrO2), cerium oxide (CeO2), magnesium oxide (MgO), zinc oxide (ZnO), and mixed-metal oxide systems, are then reviewed with emphasis on their structural characteristics, uranium retention performance, modification strategies, and immobilization mechanisms. Current evidence demonstrates that U(VI) sequestration is strongly governed by the nature and accessibility of surface hydroxyl and metal–oxygen sites, which can be further regulated through structural modification, surface functionalization, defect engineering, and the construction of mixed-metal oxide systems. This review provides a systematic understanding of the relationships among oxide composition, surface structure, interfacial chemistry, and U(VI) sequestration behavior, offering guidance for the rational design of advanced metal oxide-based uranium sequestration materials.

## 1. Introduction

Uranium is a naturally occurring radioactive heavy metal that has been extensively utilized in nuclear power generation and the nuclear fuel cycle. The development of nuclear energy, together with uranium mining, milling, processing, and nuclear fuel production, has increased the potential release of uranium into aquatic environments. Once introduced into water, uranium can migrate through surface-water and groundwater systems and accumulate in environmental media, raising concerns because of both its chemical toxicity and radioactivity [1]. Under oxic aqueous conditions, uranium predominantly occurs in the hexavalent state, U(VI), whereas its environmental mobility is strongly dependent on aqueous speciation and complexation with naturally occurring ligands, particularly carbonate [2]. Consequently, effective uranium sequestration from contaminated water is essential for limiting its environmental migration and potential exposure. Meanwhile, considering uranium as a valuable strategic resource for nuclear energy development, the integration of uranium removal with resource recovery has received increasing attention within the framework of sustainable resource management [3]. Therefore, advanced uranium treatment technologies are expected to achieve not only efficient uranium immobilization but also subsequent regeneration and resource recovery, enabling simultaneous environmental remediation and sustainable uranium utilization.

A variety of physicochemical technologies have been developed for the treatment of uranium-containing water, including chemical precipitation, ion exchange, membrane separation, solvent extraction, photocatalytic reduction, and adsorption [4]. Among these approaches, adsorption has attracted sustained attention because of its relatively simple operation, low energy requirement, adaptability to low uranium concentrations, and potential compatibility with adsorbent regeneration and uranium recovery [5]. Accordingly, numerous classes of adsorbents have been investigated, including carbonaceous materials, clay minerals, porous organic materials, ion-exchange materials, functional polymers, and inorganic minerals [4]. Among inorganic adsorbents, metal oxides are particularly attractive because of their chemical stability, tunable surface properties, and abundant reactive oxygen-containing sites. Their significance extends beyond engineered water treatment because Fe-, Mn-, Al-, and Ti-containing oxides are also common mineral components in soils and sediments, where interactions at oxide–water interfaces can substantially influence uranium retention and environmental mobility [6,7].

The retention behavior of uranium on metal oxides is closely associated with the physicochemical characteristics of the oxide-water interface. Hydrated oxide surfaces contain reactive metal-oxygen and surface hydroxyl sites that can directly interact with uranyl species. Spectroscopic and surface complexation studies have demonstrated the formation of inner-sphere U(VI) complexes on iron and manganese oxides (MnOx), while carbonate can additionally participate in ternary surface complex formation under appropriate aqueous conditions [6,8]. Beyond surface chemistry, crystal structure, morphology, porosity, surface charge, and defect states can regulate the density, accessibility, and chemical reactivity of uranium-binding sites. For oxides containing variable-valence metal centers, interfacial redox reactions may further contribute to uranium immobilization. For example, spectroscopic investigations of magnetite (Fe3O4) have demonstrated that uranium can occur predominantly as adsorbed U(VI) surface species while partial reduction to U(IV) occurs under specific conditions [9]. Therefore, uranium sequestration cannot be evaluated solely in terms of bulk oxide composition or specific surface area; the chemical identity, coordination environment, and accessibility of reactive surface sites are equally important.

Considerable efforts have therefore been devoted to regulating the structures and surface properties of metal oxides to improve uranium capture. Strategies including crystal-phase regulation, morphology and porosity engineering, surface functionalization, hydroxyl enrichment, defect engineering, and the construction of mixed-metal oxides and heterogeneous interfaces have been employed to modify the number, accessibility, and chemical environment of uranium-binding sites [10,11,12,13]. These strategies have produced a broad range of Fe-, Ti-, Al-, Mn-, Zr-, Ce-, Mg-, Zn-, and multimetallic oxide retention with distinct uranium adsorption behaviors. However, differences in oxide composition and structure, surface chemistry, aqueous conditions, and experimental protocols make direct comparison among these materials difficult. Moreover, adsorption capacity alone does not adequately describe uranium sequestration by metal oxides because materials with different surface structures may immobilize uranium through substantially different interfacial interactions. Connecting the physicochemical characteristics of metal oxides with their experimentally supported uranium-binding behavior is therefore important for understanding their adsorption performance and guiding the rational development of improved uranium adsorbents.

Several previous reviews have provided valuable overviews of uranium adsorption and separation using broad classes of adsorbent materials, while others have focused on specific material families such as aerogels or iron-based adsorbents [4,13,14,15]. However, the relationships among oxide surface chemistry, structural characteristics, aqueous U(VI) speciation, and interfacial binding mechanisms have not been consistently integrated across different metal oxide systems. The distinctive contribution of the present review is therefore to analyze metal oxide-based U(VI) adsorption from a unified mechanism- and structure–property-oriented perspective. Particular attention is given to how the chemical identity, coordination environment, accessibility, and dynamic evolution of active surface sites regulate uranium sequestration. This perspective provides a mechanistic basis for comparing different metal oxide systems and for guiding the rational design of advanced uranium adsorbents.

Accordingly, this review provides a systematic overview of metal oxide-based materials for U(VI) sequestration from aqueous environments. The fundamental chemistry governing U(VI) sequestration is first introduced, focusing on aqueous uranium speciation, metal oxide surface chemistry, and the principal interfacial interactions responsible for uranium retention. Representative metal oxide-based materials are subsequently reviewed, including iron, titanium, aluminum, manganese, zirconium, cerium, magnesium, zinc, and mixed-metal oxide systems, with emphasis on their structural characteristics, adsorption performance, modification strategies, and experimentally supported uranium immobilization mechanisms. Finally, the remaining challenges associated with adsorption selectivity, material stability and regeneration, performance under complex aqueous conditions, and practical application are discussed, together with future opportunities for the development of efficient and robust metal oxide-based materials for uranium sequestration and recovery.

## 2. Fundamental Chemistry Governing U(VI) Sequestration on Metal Oxides

The adsorption of uranium by metal oxides is governed by the chemical states of both aqueous uranium species and oxide surfaces. In oxic aqueous environments, uranium predominantly occurs in the hexavalent state, U(VI), with the linear uranyl ion (UO22+) serving as the fundamental structural unit of most dissolved uranium species [16]. However, U(VI) speciation changes substantially with pH and ligand composition, while metal oxide surfaces simultaneously undergo protonation, deprotonation, and hydroxylation reactions [17,18]. Uranium adsorption therefore reflects the coupling between aqueous speciation and oxide surface chemistry rather than an intrinsic adsorption property of the solid alone. Understanding these fundamental interactions is essential for interpreting the markedly different adsorption behaviors of Fe-, Ti-, Al-, Mn-, and other metal oxides discussed in the following sections.

### 2.1. Aqueous Speciation of U(VI)

**Figure 1:**
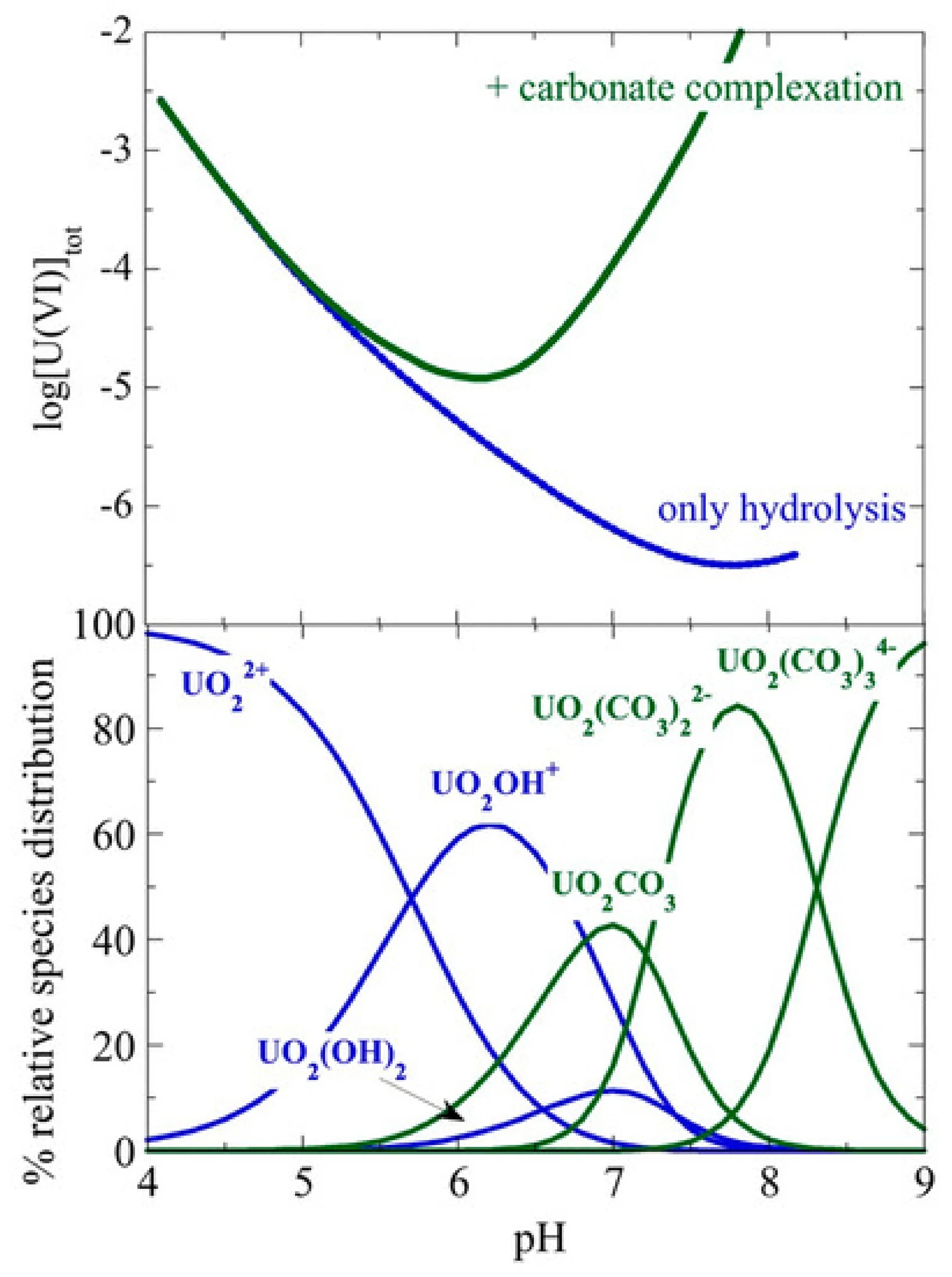
pH-dependent aqueous speciation of U(VI) and the influence of carbonate complexation on UO2(OH)2 solubility. (Adapted from Georgiou et al. [15]).

Under oxidizing conditions, dissolved U(VI) is primarily present as uranyl-based species. The UO22+ ion possesses a nearly linear O=U=O configuration, with two short axial U-O bonds and additional ligands coordinated in the equatorial plane [19]. Structural studies indicate axial U-O distances of approximately 1.77 Å for aqueous uranyl, while approximately five water molecules commonly occupy the equatorial coordination shell with average U-O distances around 2.41 Å [16]. This characteristic coordination geometry is retained in many hydrolyzed and ligand-complexed U(VI) species and strongly influences its subsequent interaction with oxygen-containing sites on metal oxide surfaces. The aqueous speciation of U(VI) is highly pH-dependent (Figure 1). Under strongly acidic conditions, free UO22^+^ constitutes an important dissolved species, while adsorption may be suppressed by competition from H^+^ and protonation of surface hydroxyl groups. With increasing pH, progressive hydrolysis produces mononuclear and polynuclear species, including UO2OH^+^, UO2(OH)2(aq), (UO2)2(OH)22+, and (UO2)3(OH)5+ [15,20]. At the same time, deprotonation of oxide surfaces generally increases the availability of reactive surface sites. The combined evolution of aqueous U(VI) speciation and surface charge therefore commonly leads to enhanced adsorption from strongly acidic to weakly acidic or near-neutral conditions. Carbonate introduces an additional level of complexity that is particularly relevant to natural groundwater and seawater. At circumneutral and alkaline pH, dissolved carbonate can form highly stable complexes with U(VI), including UO2CO3(aq), UO2(CO3)22-, and UO2(CO3)34- [21]. Formation of these neutral or negatively charged species can inhibit adsorption by stabilizing U(VI) in solution and decreasing the fraction of uranyl and hydrolyzed species available for direct surface complexation; negatively charged uranyl–carbonate complexes may also experience unfavorable electrostatic interactions with deprotonated oxide surfaces. However, carbonate is not exclusively inhibitory. Under appropriate conditions, carbonate can also participate in ternary surface complex formation. For example, uranyl-carbonate-containing surface species have been identified on ferrihydrite and MnO2 [7,22]. Therefore, the net effect of carbonate reflects the balance between aqueous uranyl–carbonate complexation and interfacial complex formation and depends on pH, dissolved inorganic carbon concentration, oxide surface chemistry, and uranium loading.

Beyond carbonate, other inorganic ions and natural organic matter (NOM) commonly present in aquatic environments can further regulate U(VI) adsorption on metal oxides. Divalent cations such as Ca^2+^ and Mg^2+^ may influence uranium retention through competition for surface hydroxyl sites and the formation of aqueous uranyl–cation–carbonate complexes. For example, Dong and Brooks demonstrated that alkaline earth metals, particularly Ca^2+^ and Mg^2+^, can form stable ternary uranyl–carbonate complexes, thereby modifying U(VI) aqueous speciation under carbonate-rich conditions [23]. In contrast, phosphate generally exhibits a strong affinity toward metal oxide surfaces and can either compete with U(VI) for reactive sites or enhance uranium immobilization through the formation of phosphate-associated surface complexes and secondary uranium phosphate phases. Studies on goethite systems have shown that phosphate can significantly modify U(VI) adsorption behavior by altering surface coordination environments and promoting the formation of stable uranium-phosphate species [24]. Sulfate and silicate may also affect U(VI) retention by competing for surface sites or modifying mineral surface properties, although their effects strongly depend on oxide mineralogy, pH, and solution composition. NOM introduces further complexity because oxygen-containing functional groups (e.g., carboxyl and phenolic groups) can directly complex U(VI), while NOM adsorption onto oxide surfaces may block reactive sites or generate additional binding environments [25]. Therefore, adsorption capacities obtained from simplified laboratory systems should be interpreted cautiously when predicting U(VI) behavior in chemically complex natural waters.

Other inorganic and organic ligands, including phosphate, silicate, sulfate, and natural organic matter, may similarly modify U(VI) speciation and its affinity toward oxide surfaces. Their effects can be competitive or cooperative depending on whether they stabilize dissolved uranium complexes, occupy surface sites, or participate in ternary interfacial structures [16,26,27,28]. Therefore, aqueous U(VI) speciation provides the chemical starting point for understanding uranium adsorption, while the ultimate immobilization pathway is determined by its interaction with the physicochemical environment of the oxide surface.

### 2.2. Surface Chemistry of Metal Oxides

Metal oxide surfaces undergo hydration and hydroxylation when exposed to water, resulting in the formation of surface hydroxyl groups generally represented as ≡M-OH, where M denotes the surface metal center. These hydroxyl groups constitute some of the most important reactive sites for uranium adsorption. Their protonation state varies with solution pH and can be conceptually described by the equilibria:

(1) $$\equiv M\text{-}\mathrm{OH}+H^{+}⇌\;\equiv M\text{-}{\mathrm{OH}}_{2}^{\;+}\;$$

(2) $$\equiv M\text{-}\mathrm{OH}⇌\;\equiv M\text{-}O^{-}+H^{+}$$

Accordingly, the surface charge of a metal oxide is not constant but varies with pH. Below the point of zero charge (pHpzc), protonated surface sites are favored and the oxide surface tends to carry a net positive charge, whereas deprotonation becomes increasingly important above pH_p_zc, producing a more negatively charged surface [29]. The resulting electrostatic environment influences the approach of charged uranium species toward the solid–water interface. Changes in surface charge following U(VI) uptake can also provide indirect evidence for specific interfacial complexation; for example, measurable shifts in ζ-potential have been observed after U(VI) adsorption onto MnO2 [7].

However, uranium affinity cannot be explained solely by electrostatic attraction. Surface hydroxyl groups also function as chemically specific coordination sites capable of replacing water or other ligands in the equatorial coordination shell of uranyl. Consequently, two oxides with comparable surface areas may exhibit substantially different U(VI) adsorption because the density, acidity, accessibility, and local coordination geometry of their surface hydroxyl groups differ. This distinction is directly relevant to the oxide systems discussed later: Ti-OH, Al-OH, Zr-OH, and Ce-OH sites provide chemically different environments for uranium coordination, while Mn-O and Fe-O(H) sites exhibit additional structural and, in some cases, redox characteristics. Crystal structure and surface termination further regulate the distribution and reactivity of these sites. Different polymorphs of the same oxide can expose metal and oxygen atoms with different coordination numbers, resulting in variations in hydroxyl density and binding geometry. Likewise, nanoscale morphology and porosity can alter the proportion of exposed reactive sites and their accessibility to aqueous uranium species. Thus, specific surface area primarily describes the amount of available interface, whereas the chemical nature of the exposed sites determines how strongly uranium interacts with that interface.

## 3. Mechanisms of Uranium Removal by Metal Oxide-Based Materials

Uranium retention by metal oxide-based materials is not necessarily limited to surface adsorption, as additional immobilization pathways may contribute depending on mineral properties, aqueous chemistry, uranium loading, and reaction conditions. These pathways, including surface adsorption (primarily through surface complexation), surface precipitation, redox transformation, and structural incorporation, represent distinct mechanisms and should therefore be evaluated based on their specific characteristics and the level of experimental evidence available for their identification.

Once uranium species approach the hydrated oxide surface, several interfacial processes can contribute to their retention. Among these, surface complexation represents one of the most widely observed mechanisms for U(VI) adsorption on metal oxides [13]. Surface complexation involves coordination between uranium and oxygen-containing surface sites and may occur through different geometries depending on the structure of the oxide and aqueous uranium species. In inner-sphere complexes, uranium is directly coordinated to surface oxygen atoms without an intervening hydration shell. Monodentate, bidentate, and bridging configurations have been proposed or experimentally identified for different oxide surfaces [30]. A classic example is ferrihydrite, for which extended X-ray absorption fine structure (EXAFS) supported surface complexation modeling identified an inner-sphere mononuclear bidentate U(VI) complex involving Fe-O surface sites [22]. Similar inner-sphere coordination has subsequently been demonstrated for other oxide systems, including MnO2 [7].

Electrostatic interactions also influence uranium uptake, particularly by controlling the initial attraction or repulsion between aqueous uranium species and charged oxide surfaces. Their contribution is strongly pH-dependent because both uranium speciation and oxide surface charge change simultaneously [31]. At strongly acidic pH, protonation of oxide surfaces and competition from H+ can suppress adsorption of cationic U(VI) species. As pH increases, deprotonation of surface hydroxyl groups generally favors the interaction of positively charged uranyl species with oxide surfaces [32]. At higher pH in carbonate-containing systems, however, the formation of negatively charged uranyl-carbonate complexes may weaken this favorable electrostatic interaction. Electrostatic attraction should therefore be regarded as one contribution to uranium adsorption rather than sufficient evidence for the molecular binding mechanism.

At elevated uranium surface loading or under solution conditions favorable for U(VI) hydrolysis, surface precipitation may contribute increasingly to uranium retention. This process differs from conventional surface adsorption because uranium-containing surface-associated or solid phases form after accumulation of U(VI) at the interface. Consequently, a high adsorption capacity or rapid uranium removal alone should not be regarded as evidence for precipitation. Stronger support requires spectroscopic or structural evidence for the formation of U-containing precipitated phases or a clear transition from adsorbed surface complexes to surface-associated precipitates. Previous studies on γ-Al2O3 and MnO2 have demonstrated that the relative contribution of surface complexation and precipitation can change with pH, uranium concentration, and surface loading [33,34,35,36].

For oxides containing variable-valence metal centers, electron-transfer reactions may provide an additional uranium immobilization pathway. Fe(II)-bearing phases are particularly relevant because interfacial electron transfer can promote the transformation of relatively mobile U(VI) into less soluble U(IV)-containing species [37]. Nevertheless, the presence of a redox-active metal alone does not demonstrate that uranium reduction has occurred. Identification of U(VI)-to-U(IV) transformation requires direct evidence from techniques capable of resolving uranium oxidation state, such as X-ray Photoelectron Spectroscopy (XPS) or X-ray absorption spectroscopy (XAS). This distinction is important when comparing Fe- and Mn-containing materials with non-redox-active oxides such as Al2O3, where uranium immobilization is predominantly controlled by coordination and precipitation processes rather than valence transformation.

Structural incorporation is distinct from surface adsorption and precipitation because uranium is accommodated within lattice sites of the host mineral or a newly formed mineral phase. This pathway has been proposed for iron-bearing minerals undergoing mineral transformation and related processes [38]. Evidence based solely on atomistic simulations or structural models establishes the plausibility of particular incorporation configurations, but confirmation of actual lattice incorporation requires direct structural characterization, preferably supported by complementary spectroscopy.

Overall, U(VI) sequestration on metal oxides arises from the coupling of aqueous uranium speciation, surface hydroxyl chemistry, surface charge, and specific interfacial reactions. Surface complexation constitutes a central immobilization pathway for many oxide systems, whereas electrostatic interactions regulate uranium accessibility to reactive sites and surface precipitation or redox transformation may become important under specific chemical conditions. These processes represent distinct retention pathways and require different levels of experimental evidence for confirmation.

**Figure 2:**
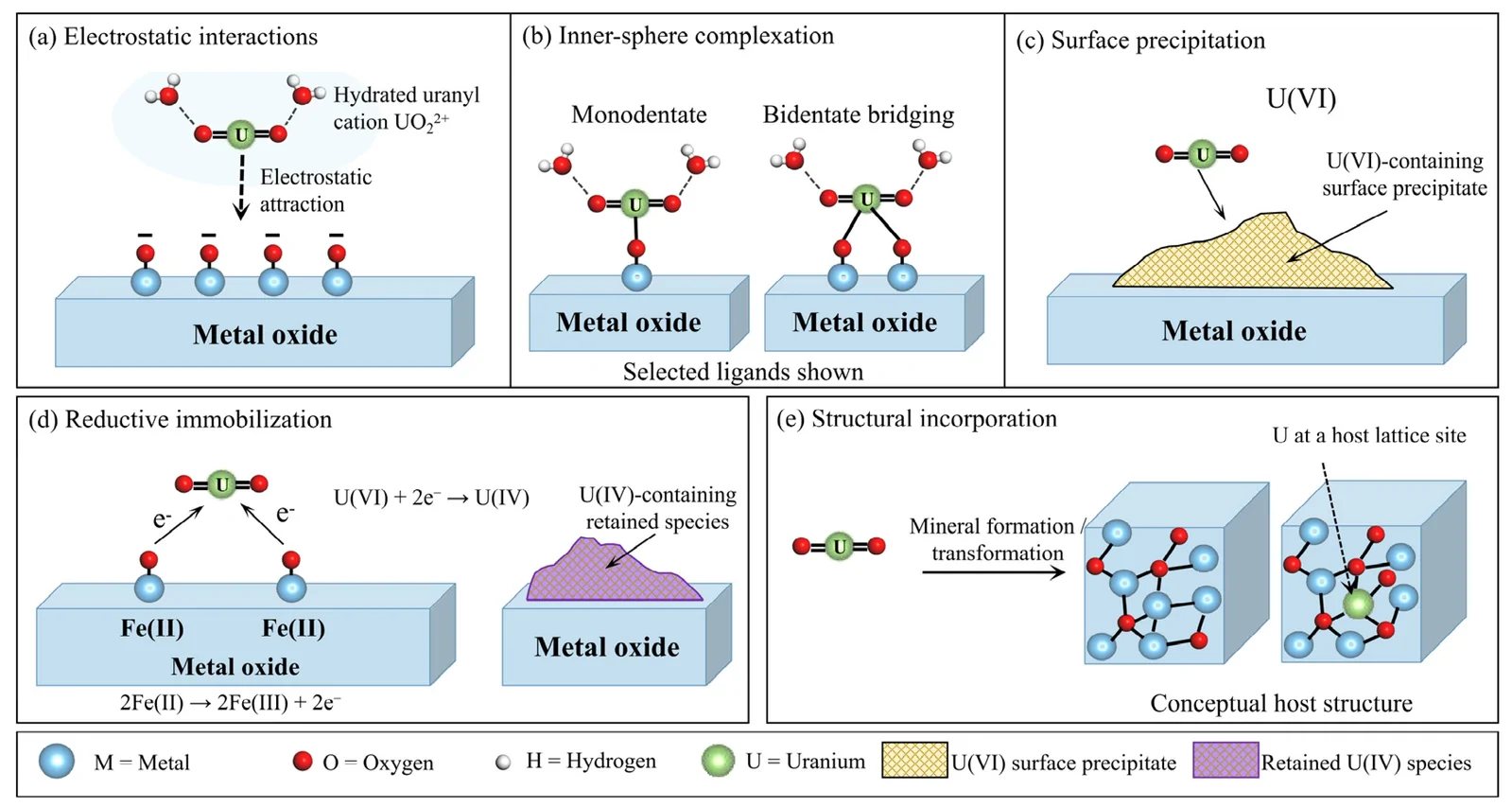
Schematic illustration of the principal uranium-retention mechanisms associated with metal oxide-based materials. (**a**) electrostatic interactions, (**b**) inner-sphere complexation, (**c**) surface precipitation, (**d**) reductive immobilization and (**e**) structural incorporation.

These pathways are summarized schematically in Figure 2 and provide a common framework for interpreting the material-specific uranium retention behaviors discussed in the following sections.

## 4. Metal Oxides

Metal oxides represent one of the most extensively investigated classes of inorganic materials for uranium removal because of their high chemical stability, widespread availability, and abundant surface-active sites. As important components of natural rocks, soils, and sediments, metal oxides also play a critical role in controlling the migration, transformation, and retention of uranium in environmental systems [14]. Representative metal oxides used for uranium adsorption include iron oxides, titanium dioxide (TiO2), Al2O3, and MnOx. Their uranium adsorption behavior is closely associated with their crystal structures, surface hydroxyl groups, surface charge characteristics, and, in some cases, variable metal valence states. Accordingly, U(VI) uptake by metal oxides may involve multiple processes, including surface complexation, electrostatic interaction, surface precipitation, and redox-assisted immobilization, with the dominant pathway varying among different oxide systems and aqueous conditions. Although pristine metal oxides often exhibit limited adsorption capacity or selectivity, considerable improvements can be achieved through crystal-phase regulation, morphology engineering, surface functionalization, and composite construction. These strategies increase the number and accessibility of active sites while simultaneously regulating the chemical interactions between uranium species and oxide surfaces.

### 4.1. General Synthesis Strategies and Structural Regulation of Metal Oxides

The structural regulation of metal oxides is essential for optimizing their uranium sequestration performance, as synthesis processes determine the crystal phase, morphology, porosity, surface defects, and chemical characteristics of the resulting materials. Among various preparation strategies, precipitation-based methods and hydrothermal synthesis are among the most frequently employed approaches for constructing metal oxide-based uranium sequestration materials, while other methods provide additional opportunities for controlling specific structural features.

Precipitation and coprecipitation methods are among the most widely used strategies for preparing metal oxide materials due to their simple operation, low cost, scalability, and compositional flexibility [39,40]. In these processes, metal ions are converted into hydroxide, carbonate, or other precursor phases through controlled precipitation, followed by aging and/or thermal treatment to obtain the corresponding oxide materials. By adjusting synthesis parameters such as precursor concentration, pH, precipitation rate, aging time, and calcination conditions, the phase composition, particle size, crystallinity, surface hydroxyl characteristics, and defect structures can be effectively controlled. In particular, coprecipitation provides an effective route for preparing mixed-metal oxides because multiple metal species can be simultaneously incorporated during precursor formation, facilitating the generation of relatively homogeneous compositions and diverse metal–oxygen coordination environments. Therefore, precipitation-based strategies are frequently adopted for the synthesis of Fe-, Mn-, Al-based oxides and mixed-metal oxide systems applied for uranium sequestration.

Hydrothermal and solvothermal methods are also extensively applied for synthesizing metal oxides with controlled morphologies and hierarchical structures [41,42]. Under elevated temperature and pressure conditions, precursor transformation and crystal growth can be effectively regulated through reaction parameters such as solvent composition, temperature, and reaction time. These approaches enable the formation of nanostructures including nanosheets, nanorods, hollow structures, and porous architectures, which can improve the exposure and accessibility of uranium-binding sites. For uranium sequestration, such structural advantages are particularly important because the accessibility and local chemical environment of surface sites directly influence uranyl coordination and immobilization behavior.

Other synthesis strategies, including sol–gel, thermal treatment/calcination, and template-assisted methods, provide additional approaches for regulating the structural and surface properties of metal oxides. Sol–gel methods enable homogeneous mixing of metal precursors and allow the regulation of oxide composition and surface chemical characteristics through hydrolysis and condensation processes [43]. Thermal treatment and calcination are commonly employed to convert precursor materials into crystalline oxide phases while influencing crystallinity, defect structures, and oxygen-containing species [44,45]. In addition, template-assisted and morphology-controlled strategies can be used to construct porous and hierarchical oxide architectures with improved accessibility of uranium-binding sites. For mixed-metal oxides, compositional regulation during synthesis further enables the generation of heterogeneous interfaces and diverse metal–oxygen coordination environments, which may influence uranium immobilization pathways.

Overall, synthesis strategies serve not only as routes for obtaining desired oxide phases but also as important tools for regulating structural characteristics and interfacial properties that govern U(VI) sequestration behavior.

### 4.2. Iron Oxides

Iron oxides are among the most extensively studied inorganic materials for uranium removal owing to their widespread occurrence, abundant surface hydroxyl groups, variable Fe valence states, and strong affinity toward uranium species. In natural environments, iron-bearing minerals participate extensively in the migration, transformation, and immobilization of uranium [14]. Representative iron-based materials investigated for uranium removal include hematite (α-Fe2O3), maghemite (γ-Fe2O3), Fe3O4, goethite (α-FeOOH), and nanoscale zero-valent iron (nZVI). Their uranium-removal behavior is strongly dependent on the surface chemistry and redox properties of the specific iron-bearing phase. Surface hydroxyl groups can provide coordination sites for U(VI), whereas Fe(II)-containing or zero-valent iron systems may additionally induce electron-transfer processes. Consequently, uranium removal by iron-based materials can involve surface complexation, reduction, coprecipitation, mineralization, and even incorporation into newly formed mineral phases rather than a single adsorption pathway.

The redox activity of iron-based materials provides an important route for enhancing uranium immobilization. In reducing systems, soluble U(VI) can accept electrons from Fe(0) or Fe(II)-containing species and be converted into less soluble U(IV), followed by its retention through precipitation, coprecipitation, or association with iron mineral phases. Zhang et al. [46] prepared magnesium hydroxide-coated nanoscale zero-valent iron composites for uranium-containing wastewater treatment, in which uranium removal was achieved through the synergistic contribution of adsorption and reduction [47]. In such systems, surface adsorption facilitates the enrichment of U(VI) near reactive iron sites, while subsequent electron transfer promotes its transformation into less mobile uranium species. This adsorption–reduction coupling can substantially enhance uranium immobilization compared with surface adsorption alone. Nevertheless, the stability of reductively immobilized uranium remains dependent on environmental conditions. Dissolved oxygen and other oxidizing species may induce the reoxidation of U(IV), potentially resulting in uranium remobilization. Therefore, the long-term stability of reduced uranium species is an important consideration when applying redox-active iron materials to environmental remediation.

**Figure 3:**
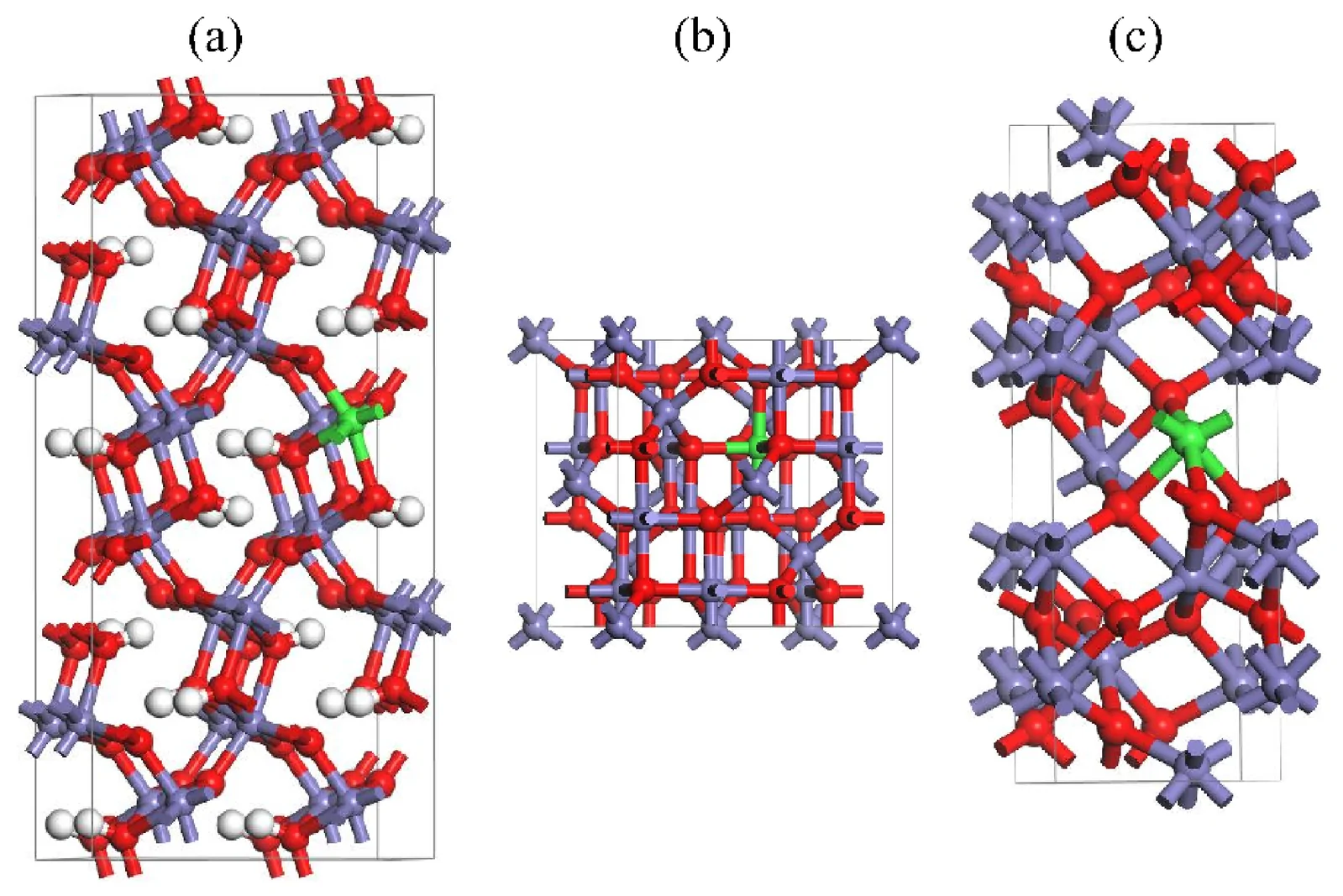
Representative structural configurations of uranium incorporation into iron-bearing minerals, including (**a**) α-FeOOH, (**b**) Fe3O4, and (**c**) α-Fe2O3. Iron atoms are shown in purple, oxygen atoms are in red and hydrogen atoms are in white. The uranium incorporation sites are shown in green.

In addition to surface retention and reductive immobilization, incorporation of uranium into iron-bearing mineral structures provides another pathway for long-term stabilization. Uranium species can become structurally associated with iron oxide phases during hydrothermal treatment, coprecipitation, mineral transformation, or related processes [38]. α-FeOOH, α-Fe2O3, and Fe3O4 have been investigated as representative mineral hosts, and structural simulations have further demonstrated the possible incorporation configurations of uranium in iron-bearing mineral structures (Figure 3). Unlike relatively reversible surface adsorption, structural incorporation can restrict uranium mobility within the mineral lattice and therefore potentially provide greater resistance to environmental perturbations. However, lattice incorporation should be distinguished from conventional adsorption because it generally involves mineral formation or structural transformation rather than simple occupation of surface adsorption sites. From the perspective of uranium remediation, these processes illustrate the diverse pathways through which iron-bearing materials can transform initially adsorbed uranium into more stable immobilized forms.

**Figure 4:**
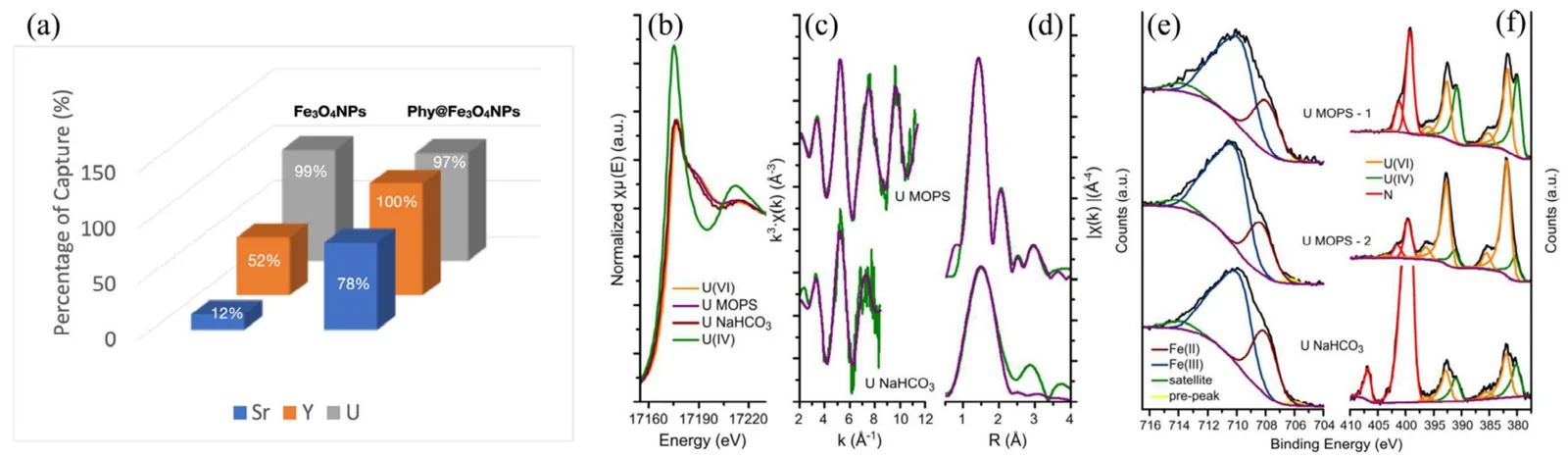
U(VI) uptake and spectroscopic characterization of uranium interactions with Fe3O4. (**a**) Capture efficiencies of pristine Fe3O4 and phytate-coated Fe3O4 nanoparticles toward U(VI), Y(III), and Sr(II) under competitive conditions. (Adapted from Duel et al. [48]). (**b**–**d**) U LIII-edge XANES and EXAFS spectra and corresponding Fourier-transformed EXAFS data for U(VI) associated with the Fe3O4 (111) surface. (**e**,**f**) Fe 2p and U 4f XPS spectra showing the oxidation-state distributions of Fe and U, respectively. (Adapted from Bots et al. [9]).

Magnetic iron oxides are particularly attractive for practical uranium removal because they combine high surface reactivity with convenient magnetic separation. Among them, Fe3O4 has been extensively investigated owing to its strong magnetic response and the coexistence of Fe(II) and Fe(III), which provides the material with both adsorption and redox activity. A representative study demonstrated that pristine Fe3O4 nanoparticles exhibited nearly quantitative uptake of U(VI) from aqueous solution (Figure 4a) [48]. Uranium removal was initially associated with the interaction of aqueous uranyl species with the reactive Fe3O4 surface, while the Fe(II)/Fe(III) redox couple could further participate in electron-transfer processes involving U(VI)/U(IV). Therefore, unlike metal oxides dominated primarily by surface coordination, Fe3O4 can couple interfacial adsorption with redox-assisted uranium immobilization. Moreover, the intrinsic magnetic properties of Fe3O4 enable rapid recovery of the uranium-loaded particles using an external magnetic field, providing an additional advantage for solid–liquid separation in practical treatment systems.

The interaction between U(VI) and Fe3O4 further demonstrates that uranium uptake by iron oxides involves chemical interactions rather than simple physical adsorption. Surface-sensitive spectroscopic analyses using grazing-incidence X-ray absorption spectroscopy (GI-XAS) and XPS revealed that uranium retained on the Fe3O4(111) surface was predominantly present as U(VI) surface complexes (Figure 4b–f). EXAFS analysis further supported the formation of inner-sphere uranyl complexes at the Fe3O4 surface. Meanwhile, partial reduction of U(VI) to U(IV) could also occur, with the extent of reduction being strongly influenced by the Fe(II)/Fe(III) stoichiometry of Fe3O4 and aqueous carbonate chemistry [9]. These observations indicate that Fe3O4 can provide both reactive surface sites for uranyl complexation and redox-active Fe species for uranium transformation. Consequently, uranium immobilization on Fe3O4 may proceed through the coupling of surface complexation and redox processes, with their relative contributions depending strongly on the physicochemical conditions of the aqueous system.

Although pristine magnetic iron oxides provide reactive surfaces and convenient magnetic recovery, their adsorption capacity and selectivity can be further improved by introducing uranium-affinitive functional groups. Surface functionalization therefore represents an important strategy for regulating the interfacial chemistry of magnetic iron oxide adsorbents. For example, maleic anhydride-β-cyclodextrin-functionalized magnetic iron oxide nanoparticles (IONPs@MAH-β-CD) exhibited a maximum Langmuir adsorption capacity of 163.93 mg·g^−1^, substantially higher than that of the corresponding unmodified magnetic nanoparticles [49]. The functional coating introduced additional oxygen-containing binding sites while preserving the magnetic response of the iron oxide core, thereby integrating enhanced uranium affinity with convenient solid–liquid separation. FTIR and XPS analyses indicated that these surface functional groups participated directly in U(VI) binding, demonstrating that surface functionalization can shift the uranium-capture behavior of magnetic iron oxides from predominantly mineral-surface interactions toward stronger and more specific coordination at engineered interfacial sites.

### 4.3. Titanium Dioxide

TiO2 is another representative metal oxide extensively investigated for uranium adsorption because of its excellent chemical stability, corrosion resistance, low toxicity, and abundant surface hydroxyl groups [50]. TiO2 mainly exists in three crystalline phases, namely anatase, rutile, and brookite [51], among which anatase is the most widely investigated in aqueous adsorption systems. At the nanoscale, the high proportion of surface atoms and hydroxylated surface provides TiO2 with favorable interfacial reactivity toward various aqueous contaminants, including Pb(II) [52,53], Cr(VI) [54,55,56], Hg(II) [57,58], and U(VI) [59,60,61]. For uranium adsorption, surface hydroxyl groups are particularly important because they provide reactive sites for the binding of aqueous uranyl species. Depending on solution chemistry and surface charge, U(VI) uptake can involve electrostatic attraction and, more importantly, the formation of inner-sphere surface complexes through Ti-OH sites. Under specific aqueous conditions, changes in uranium speciation may also promote surface-associated precipitation.

Despite these favorable properties, pristine TiO2 generally exhibits moderate U(VI) adsorption capacity because its adsorption behavior is mainly governed by the limited density and accessibility of surface binding sites [62]. To overcome this limitation, considerable attention has been devoted to tailoring the surface chemistry and structural features of TiO2. In general, current modification strategies can be categorized into two complementary approaches: introducing surface functionalities with specific coordination affinity toward uranyl species and constructing porous or hierarchical architectures to expose more accessible adsorption sites. These strategies demonstrate that enhanced U(VI) adsorption on TiO2 relies on the synergistic optimization of both the textural properties and surface chemical environment, rather than simply increasing surface area or pore volume.

Surface functionalization with organic polymers provides an effective approach for introducing additional coordination sites onto TiO2. For example, polyacrylamide/TiO2(PAM/TiO2) composites have been developed for the removal of U(VI) from aqueous solutions [63]. The composite exhibited a maximum Langmuir adsorption capacity of 146.5 mg·g^−1^, while XPS analysis indicated that uranium uptake was mainly associated with electrostatic interactions and the participation of amide groups introduced by polyacrylamide. In this system, polymer functionalization therefore provides additional chemically active sites beyond the intrinsic Ti-OH groups of TiO2, enabling stronger interfacial interactions with aqueous uranium species. This example demonstrates that the adsorption performance of TiO2 can be enhanced not only by increasing surface area but also by deliberately tailoring the chemical nature of uranium-binding sites.

**Figure 5:**
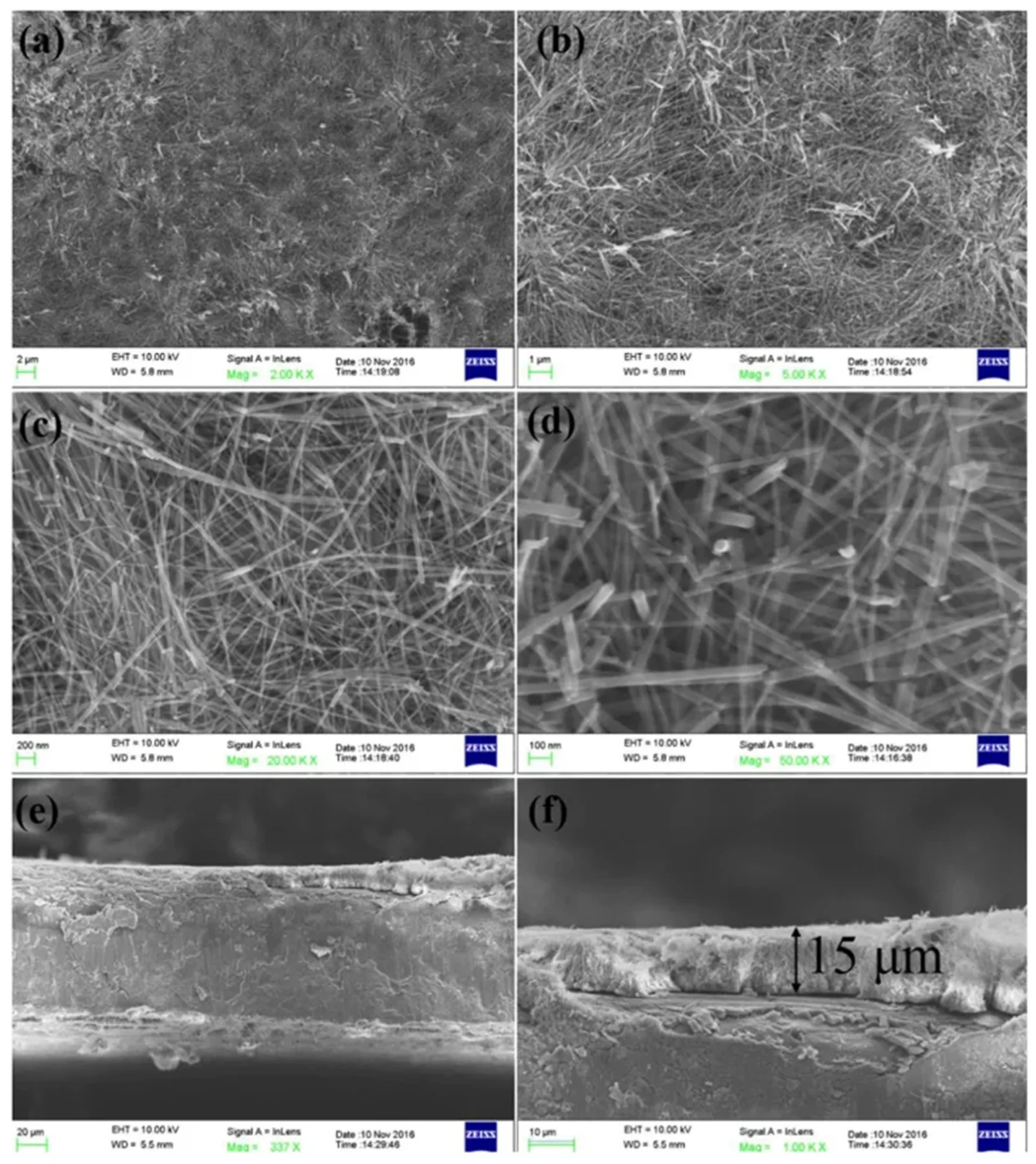
(**a**–**f**) SEM images of TiO2 nanowire arrays in situ grown on Ti foil (TiO2/Ti). (Adapted from Chen et al. [64]).

In addition to surface functionalization, morphology engineering provides another effective strategy for improving uranium adsorption by increasing the exposure and accessibility of TiO2 active sites. Chen et al. [64] constructed TiO2 nanowire arrays directly on Ti foil, producing an integrated TiO2/Ti adsorbent with highly exposed one-dimensional nanostructures. The material exhibited efficient U(VI) adsorption over a relatively broad pH range of 5.0–9.0, with a maximum Langmuir adsorption capacity of 354.5 mg·g^−1^ at 323 K. The vertically distributed TiO2 nanowires provide abundant accessible solid–liquid interfaces and facilitate the transport of uranium species toward surface adsorption sites. Meanwhile, immobilization of the nanowire arrays on Ti foil enables straightforward recovery of the adsorbent after treatment, avoiding the difficult separation commonly encountered with dispersed TiO2 nanoparticles (Figure 5). This example illustrates how morphology regulation can simultaneously improve active-site accessibility and the practical recoverability of TiO2-based adsorbents.

Further enhancement of uranium adsorption can be achieved by constructing three-dimensional porous TiO2 architectures. A TiO2 aerogel prepared through a template-assisted strategy exhibited a highly developed porous network and rapid U(VI) adsorption, reaching apparent equilibrium within approximately 100 min [65]. The material achieved a maximum Langmuir adsorption capacity of 638.0 mg·g^−1^ at 298 K and maintained a uranium removal efficiency above 90% after five adsorption–desorption cycles. Compared with conventional particulate TiO2, the interconnected aerogel framework provides a larger accessible interface and facilitates diffusion of uranium species throughout the porous structure. Importantly, the enhanced performance of porous TiO2 should not be attributed to pore structure alone: the porous architecture primarily improves the accessibility of reactive surface sites, whereas the ultimate immobilization of U(VI) remains governed by chemical interactions with the TiO2 surface. The combination of high site accessibility, rapid adsorption, and favorable recyclability therefore makes porous TiO2 architectures particularly attractive for uranium removal.

Beyond improving adsorption capacity alone, TiO2 has increasingly been incorporated into multifunctional composites to integrate uranium capture with convenient material recovery. A representative example is Fe3O4@PDA@TiO2, in which the Fe3O4 component provides magnetic responsiveness, the polydopamine (PDA) interlayer contributes additional functional groups and interfacial adhesion, and the outer TiO2 phase provides chemically stable adsorption interfaces [66]. Such hierarchical composite construction enables different components to perform complementary functions, including uranium coordination, interfacial enrichment, structural stabilization, and magnetic separation. This strategy is particularly relevant to uranium extraction from complex aqueous environments, where adsorption capacity alone is insufficient and selectivity, recovery, and cyclic stability must also be considered.

Overall, the development of TiO2-based uranium adsorbents has evolved from the direct utilization of pristine TiO2 toward simultaneous regulation of surface chemistry and hierarchical structure. The intrinsic uranium affinity of TiO2 originates primarily from interactions between aqueous uranium species and hydroxylated TiO2 surfaces, whereas polymer functionalization introduces additional heteroatom-containing coordination sites and morphology engineering improves their accessibility. Further integration with magnetic and interfacial components extends these materials from simple adsorbents toward multifunctional uranium-capture systems.

### 4.4. Aluminum Oxides

Aluminum oxides are an important class of metal oxide adsorbents for uranium because of their high chemical stability, abundant surface hydroxyl groups, and tunable crystal and pore structures. In contrast to redox-active iron oxides, uranium uptake by Al2O3 generally does not involve pronounced valence transformation but is primarily governed by interfacial reactions between aqueous uranium species and hydroxylated alumina surfaces. Therefore, the density and coordination environment of surface Al-OH sites, crystal structure, pore accessibility, and aqueous uranium speciation collectively determine the uranium adsorption behavior of Al2O3.

At the molecular scale, the interaction of U(VI) with alumina is dominated by specific surface complexation rather than nonspecific physical adsorption. Catalano et al. [67] investigated U(VI) adsorption on the (1-102) surface of α-Al2O3 using crystal truncation rod (CTR) diffraction and grazing-incidence extended X-ray absorption fine structure (GI-EXAFS) spectroscopy. The results demonstrated that U(VI) preferentially binds to singly coordinated surface oxygen sites, whereas steric and protonation effects restrict its interaction with other surface oxygen configurations. The adsorbed uranium retains the characteristic uranyl coordination structure and forms inner-sphere surface complexes through direct interaction with reactive oxygen sites on the alumina surface. These molecular-scale observations demonstrate that the adsorption affinity of Al2O3 is determined not only by the total number of surface hydroxyl groups but also by their local coordination environment and accessibility.

The coordination environment of adsorbed U(VI) can further evolve with increasing uranium accumulation at the alumina-water interface. Müller et al. [68] investigated U(VI) adsorption on γ-Al2O3 using time-resolved attenuated total reflection Fourier-transform infrared spectroscopy (ATR-FTIR) in combination with EXAFS spectroscopy. Three distinct interfacial uranium species were identified as a function of surface loading. At relatively low U(VI) surface concentrations, uranium was predominantly retained as monomeric carbonate-containing surface complexes. With increasing uranium accumulation, oligomeric surface complexes progressively formed, whereas further increases in surface loading ultimately resulted in surface precipitation. Thus, surface complexation and precipitation are not necessarily independent uranium-removal mechanisms but may represent successive stages of interfacial uranium accumulation on Al2O3.

**Figure 6:**
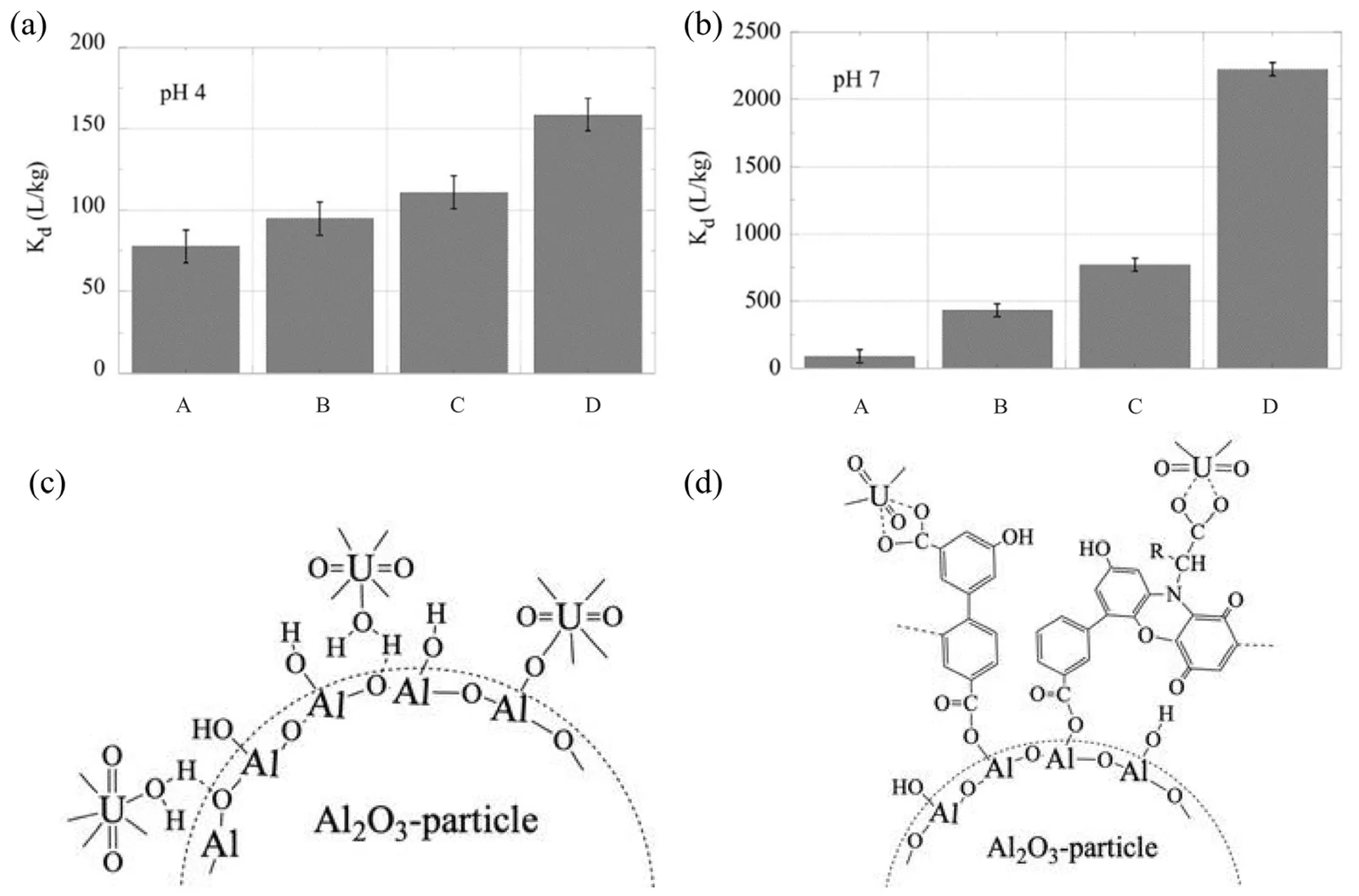
HA surface functionalization on U(VI) adsorption by Al2O3. (**a**,**b**) Distribution coefficients (Kd) for U-232 adsorption on bare and HA-treated Al2O3 particles at pH 4 and pH 7, respectively, with HA-treatment concentrations ranging from 0.01 to 1.0 g·L^−1^. (**c**,**d**) Schematic illustrations of possible U(VI) interactions with bare Al2O3 and HA-coated Al2O3 surfaces, respectively. (Adapted from Ioannidis et al. [70]).

Besides surface chemistry, structural engineering of Al2O3 provides an effective strategy for increasing the accessibility of uranium-binding sites. Liao and Zhang [69] prepared a series of porous Al2O3 materials from different aluminum precursors through a solution-freeze-drying-calcination route. The resulting materials exhibited interconnected porous structures that facilitated uranium transport toward reactive alumina surfaces. The optimized porous Al2O3 achieved a maximum Langmuir adsorption capacity of 805.72 mg·g^−1^ at 298 K and pH 7.0. Moreover, the adsorption efficiency remained above 90% after five adsorption–desorption cycles and showed favorable resistance toward coexisting ions. The enhanced uranium uptake was attributed to the porous architecture, which increased the exposure and accessibility of surface binding sites. Importantly, the role of porosity is primarily to facilitate mass transfer and expose reactive Al-OH sites, whereas uranium immobilization itself remains governed by chemical interactions at the alumina–water interface. Beyond pore-structure regulation, direct surface functionalization provides a complementary strategy for modifying the chemical nature of uranium-binding sites on Al2O3. Ioannidis et al. [70] investigated U(VI) adsorption on Al2O3 particles before and after surface modification with humic acid (HA). HA functionalization markedly enhanced the affinity of Al2O3 toward U(VI), with the enhancement being particularly pronounced under near-neutral conditions (Figure 6a,b). This behavior was attributed to the additional organic binding functionalities introduced by HA, mainly carboxyl and phenolic groups. Under near-neutral conditions, extensive deprotonation of HA carboxylic groups generates stronger U(VI)-binding sites and favors the formation of stable inner-sphere complexes (Figure 6c,d). In contrast, under more acidic conditions, protonation of the carboxylic groups weakens direct U(VI) coordination and outer-sphere interactions become more important. These results demonstrate that surface functionalization can enhance uranium uptake by changing the chemical identity and protonation state of interfacial binding sites rather than simply increasing the accessible surface area.

The adsorption behavior of Al2O3 is also highly sensitive to aqueous solution chemistry because dissolved ligands can alter both uranium speciation and its interfacial coordination structure. Mei et al. [71] investigated the influence of soluble silicate on U(VI) adsorption onto γ-Al2O3 using batch adsorption experiments and EXAFS spectroscopy. The presence of silicate significantly enhanced U(VI) adsorption, and EXAFS analysis revealed that uranium was retained through inner-sphere surface complexation. Structural fitting indicated axial and equatorial U-O coordination at approximately 1.79 and 2.43 Å, respectively, together with an Al coordination shell at approximately 3.35 Å. Silicate acted as an interfacial bridge between uranium and the γ-Al2O3 surface, promoting the formation of ternary inner-sphere surface complexes. This result demonstrates that dissolved inorganic ligands do not merely compete with uranium for adsorption sites; under appropriate conditions, they can participate directly in the construction of uranium-containing interfacial complexes and thereby enhance uranium retention.

Overall, uranium adsorption on Al2O3 is governed by a close coupling between surface-site chemistry, pore accessibility, uranium surface loading, and aqueous speciation. Reactive surface oxygen and hydroxyl sites provide the molecular basis for inner-sphere U(VI) complexation, whereas increasing surface loading can induce a transition from isolated surface complexes toward oligomeric species and ultimately surface precipitation. Porous-structure engineering increases the accessibility of these reactive sites, while dissolved ligands such as silicate can alter the coordination environment through ternary surface-complex formation. Consequently, optimization of Al2O3-based uranium adsorbents should focus not only on maximizing surface area but also on controlling the chemical nature and accessibility of surface binding sites under realistic aqueous conditions.

### 4.5. Manganese Oxides

MnOx are another important class of metal oxide materials for uranium sequestration because of their diverse crystal structures, high surface reactivity, abundant surface oxygen sites, and variable Mn oxidation states. Common manganese oxide phases include α-, β-, and δ-MnO2 as well as other MnOx structures, whose uranium adsorption behavior is strongly influenced by crystal structure, specific surface area, surface coordination environment, and aqueous chemistry [36]. Compared with metal oxides whose adsorption performance is primarily associated with surface hydroxyl density, MnOx exhibit particularly strong interactions with uranium through reactive Mn-O surface sites, making them important both as uranium adsorbents and as model minerals for understanding uranium migration in natural environments [72,73].

The crystal structure and accessible surface area of MnOx have pronounced effects on their apparent U(VI) adsorption capacities. A systematic comparison of MnOx with different structures showed that α-MnO2 exhibited particularly favorable uranium uptake, reaching a maximum Langmuir adsorption capacity of approximately 280 mg·g^−1^ [74]. Interestingly, when the adsorption capacities of different MnOx phases were normalized by their specific surface areas, their uranium uptake per unit surface area became relatively similar. This observation indicates that differences in macroscopic adsorption capacity among MnOx phases arise to a considerable extent from variations in the exposure and accessibility of reactive surfaces rather than from completely different uranium-binding chemistries. Therefore, structural regulation of MnOx can enhance uranium uptake by increasing the population of accessible Mn-O surface sites.

At the molecular scale, U(VI) retention on MnO2 is dominated by specific inner-sphere surface complexation. Wang et al. [7] systematically investigated U(VI) adsorption onto synthetic and biogenic MnO2 by combining batch adsorption experiments, ζ-potential measurements, EXAFS spectroscopy, and surface complexation modeling. Uranium adsorption was relatively insensitive to changes in ionic strength, providing evidence for specific inner-sphere interactions rather than nonspecific outer-sphere electrostatic association. EXAFS analysis and surface complexation modeling further supported the formation of monodentate and bidentate inner-sphere U(VI) surface complexes on MnO2. These results demonstrate that surface oxygen atoms of MnO2 participate directly in the coordination of uranyl species and constitute the molecular basis for the strong uranium affinity of manganese oxide surfaces.

The coordination structure of adsorbed uranium is highly dependent on aqueous conditions. Detailed ATR-FTIR and EXAFS characterization of U(VI) immobilization on α-MnO2 revealed the formation of a bidentate binuclear surface complex under mildly acidic to near-neutral conditions [74]. The asymmetric stretching vibration of the uranyl group was observed at approximately 912 cm^−1^, while EXAFS analysis identified Mn atoms in the U coordination shell, providing direct evidence for U-O-Mn interfacial bonding. At pH values of approximately 7–8, UO2(OH)2 like surface precipitates were additionally detected at the molecular scale. Consequently, uranium retention on MnO2 may evolve from inner-sphere surface complexation toward surface precipitation as uranium hydrolysis becomes increasingly favorable. This behavior again demonstrates that adsorption mechanisms on metal oxides should not be considered fixed but rather depend on uranium speciation and interfacial chemical conditions.

Dissolved inorganic carbon further influences uranium adsorption by modifying aqueous U(VI) speciation and its coordination at the MnO2 surface. Surface complexation modeling combined with EXAFS spectroscopy demonstrated that carbonate-containing systems can involve ternary uranyl-carbonato surface complexes [7]. At elevated dissolved inorganic carbon concentrations, however, stable aqueous uranyl-carbonate complexes may compete with MnO2 surfaces and suppress overall uranium adsorption. Thus, carbonate can exert apparently contrasting effects: it may participate in ternary surface-complex formation while simultaneously stabilizing soluble uranium species. The balance between these processes is controlled by pH, carbonate concentration, and uranium loading.

**Figure 7:**
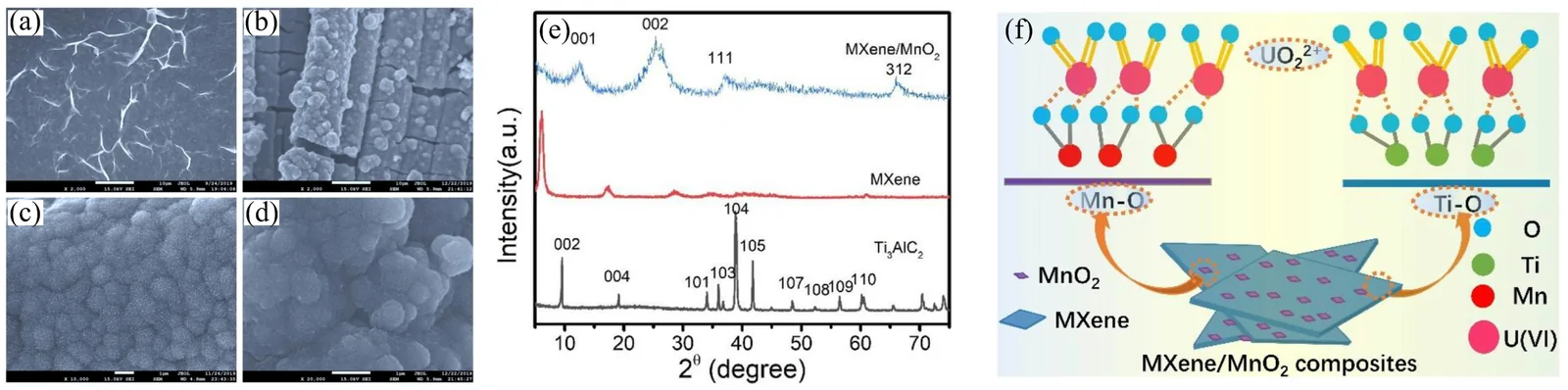
Morphological and structural characterization and proposed U(VI) removal mechanism of the MXene/MnO2 composite. (**a**) SEM image of Ti3C2 nanosheets. (**b**–**d**) SEM images of the MXene/MnO2 composite at different magnifications. (**e**) XRD patterns of Ti3AlC2, MXene, and the MXene/MnO2 composite. (**f**) Proposed mechanism for U(VI) removal by the MXene/MnO2 composite. (Adapted from Wang and Liu [75]).

Beyond pristine MnOx, composite engineering provides an effective approach for increasing active-site accessibility and integrating multiple uranium-removal pathways. A representative example is the MXene/MnO2 nanocomposite, in which MnO2 nanoparticles were coupled with a two-dimensional MXene substrate [75]. The resulting composite achieved a uranium removal efficiency of approximately 99% and an experimentally determined adsorption capacity of 696 mg·g^−1^. Structural characterization showed that MnO2 was successfully integrated with the layered MXene framework, producing abundant accessible interfaces for uranium capture (Figure 7a–e). More importantly, uranium removal involved not only interfacial adsorption but also reduction and subsequent fixation, indicating that coupling MnO2 with a redox-active substrate can extend uranium removal beyond conventional surface complexation (Figure 7f). The composite also maintained substantial uranium removal in the presence of competing ions and HA, highlighting the potential of interfacial engineering for complex aqueous systems.

Overall, MnOx exhibit strong uranium affinity primarily because of specific interactions between uranyl species and reactive Mn-O surface sites. Inner-sphere monodentate and bidentate complexes constitute important molecular configurations for adsorbed U(VI), while increasing pH and uranium loading can promote the formation of surface-associated precipitates. Meanwhile, carbonate and other dissolved ligands regulate uranium adsorption by modifying both aqueous speciation and interfacial coordination. Structural and composite engineering can further increase the accessibility of MnOx active sites and introduce complementary immobilization pathways.

### 4.6. Other Metal Oxides

Other metal oxides, including zirconium dioxide (ZrO2), cerium dioxide (CeO2), magnesium oxide (MgO), and ZnO, have also been explored for uranium sequestration because of their distinct surface chemistry, structural stability, and tunable hydroxyl, defect, and morphological characteristics. Although these materials have been investigated less extensively than Fe-, Ti-, Al-, and Mn-based oxides, they provide complementary examples of how uranium affinity can be regulated through surface functionalization, hydroxyl engineering, phase transformation, and morphology control. Their adsorption behavior further demonstrates that the uranium-capture performance of metal oxides depends not simply on specific surface area but more fundamentally on the chemical identity and accessibility of reactive surface sites.

**Figure 8:**
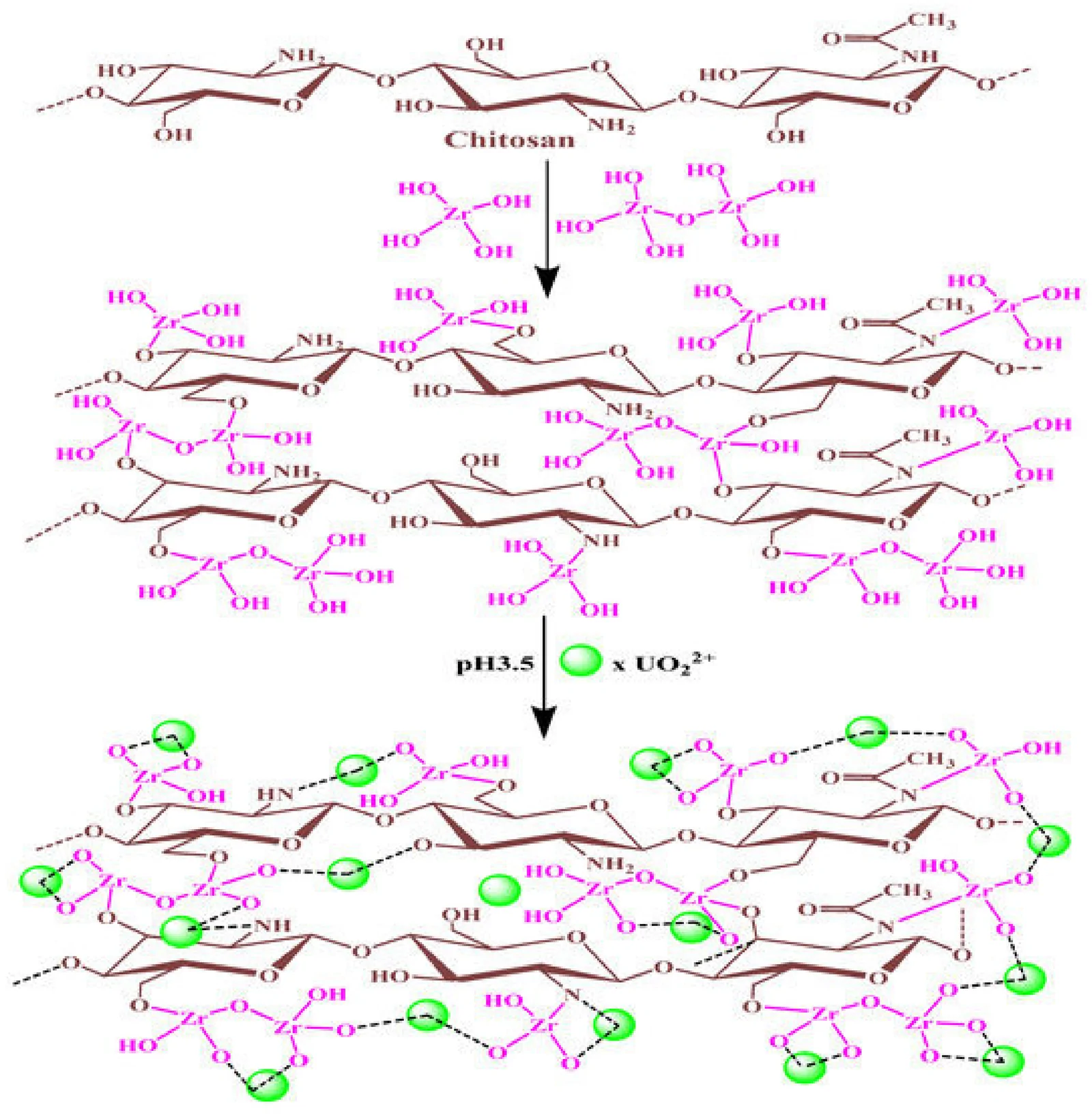
Proposed interactions between U(VI) and the ZrO2/CS composite, illustrating the participation of Zr-OH and nitrogen-containing functional groups in uranium capture. (Adapted from Negm et al. [76]).

ZrO2 is particularly attractive for uranium adsorption because of its high chemical stability and the strong affinity of hydroxylated zirconium sites toward uranyl species. However, pristine ZrO2 generally provides limited numbers of highly selective binding sites, making surface functionalization an effective strategy for improving its adsorption performance. Negm et al. [76] constructed a ZrO2/chitosan (ZrO2/CS) composite in which inorganic ZrO2 was integrated with nitrogen-containing functional groups from CS. The resulting material exhibited an experimentally determined U(VI) adsorption capacity of approximately 175 mg·g^−1^ and could be reused for seven adsorption–desorption cycles with only a limited decline in performance. Spectroscopic characterization indicated that uranium capture by ZrO2/CS was associated with the participation of Zr-OH, -NH, and -NH2 groups, which provided multiple oxygen- and nitrogen-containing coordination sites for U(VI). The authors proposed that uranium retention involved both electrostatic attraction and complexation with these surface functionalities (Figure 8).

Furthermore, ZrO2/CS was applied to uranium-containing leachate, and the adsorbed uranium was recovered through H2SO4 desorption followed by precipitation as sodium diuranate with a purity of 94.88%. This result demonstrates that the integration of inorganic oxide stability and organic coordination sites can not only enhance uranium capture but also facilitate uranium recovery from practical leaching solutions. This example illustrates an important strategy for ZrO2-based adsorbents: rather than relying solely on the intrinsic hydroxyl groups of the oxide, organic functionalization creates a chemically heterogeneous interface containing multiple uranium-binding sites, thereby improving adsorption capacity and recovery efficiency.

CeO2 provides another distinctive platform for uranium adsorption because its surface chemistry can be deliberately regulated through hydroxylation and defect engineering. In particular, the density and chemical state of surface Ce-OH groups strongly influence the affinity of CeO2 toward aqueous U(VI). Zheng et al. [12] constructed hollow CeO2 spheres rich in Ce-OH groups through an alkali-etching strategy. The resulting CeO2 reached adsorption equilibrium within only 10 min and exhibited a maximum experimentally determined U(VI) adsorption capacity of 143.1 mg·g^−1^. Moreover, uranium adsorption was only weakly affected by common competing anions and most cations, indicating good tolerance toward complex aqueous matrices. More importantly, the material enriched uranium from seawater by 167.9 times with a recovery efficiency of 83.9%, demonstrating its potential applicability for uranium recovery under environmentally relevant conditions. Mechanistic analysis showed that the enhanced U(VI) affinity was mainly associated with the abundant Ce-OH groups generated on the CeO2 surface. These hydroxyl groups served as direct uranium-binding sites, and adsorbed U(VI) was predominantly immobilized through a single-bridge coordination configuration. Importantly, this work demonstrates that increasing oxide surface area alone is not necessarily sufficient for improving uranium adsorption; deliberate construction of chemically effective hydroxyl sites can directly regulate the coordination environment and adsorption kinetics of U(VI). Therefore, surface hydroxyl engineering represents a particularly promising strategy for optimizing CeO2-based uranium adsorbents.

MgO exhibits a fundamentally different uranium-removal behavior because the oxide itself can undergo substantial structural transformation in aqueous environments. Rather than functioning exclusively as a chemically unchanged adsorption surface, MgO can hydrate to form Mg(OH)2, generating a hydroxyl-rich interfacial phase with a strong affinity toward uranium. Hierarchical mesoporous–macroporous MgO has been reported to transform into porous Mg(OH)2 upon contact with aqueous solution while partly retaining the interconnected architecture of its oxide precursor [77]. The transformed material exhibited extremely high uranium uptake under high-concentration experimental conditions, demonstrating the importance of hydration-induced surface reconstruction in uranium immobilization. Therefore, uranium removal in this system is not solely governed by the pristine MgO surface. Hydration of MgO generates a hydroxyl-rich Mg(OH)2 phase that provides additional reactive sites for uranium immobilization. This phase transformation plays an important role in the high uranium uptake and demonstrates that the adsorption retention of reactive metal oxides should be interpreted on the basis of their actual surface state under aqueous conditions.

ZnO, in contrast, illustrates the importance of morphology engineering and macroscopic material configuration. Zhang et al. [78] grew ZnO nanorod arrays directly on cotton cloth (CC) to construct an integrated ZnO adsorbent for U(VI) removal. The ZnO/CC material exhibited a maximum Langmuir adsorption capacity of 431.03 mg·g^−1^ at pH 5.0 and 298 K, while the adsorption kinetics were well described by the pseudo-second-order model. The nanorod-array architecture provided abundant accessible solid–liquid interfaces for uranium uptake, while immobilization of ZnO on a macroscopic cotton substrate facilitated recovery of the adsorbent after treatment. Thus, this system demonstrates that morphology regulation can simultaneously enhance the accessibility of oxide surface sites and address the practical separation problems commonly associated with dispersed oxide nanoparticles.

Taken together, these less extensively investigated oxides demonstrate several distinct routes for regulating uranium sequestration. ZrO2 illustrates how organic functionalization can introduce complementary coordination sites at an oxide interface; CeO2 demonstrates the direct regulation of U(VI) affinity through surface hydroxyl engineering; MgO highlights dynamic phase transformation and the generation of reactive hydroxide surfaces under aqueous conditions; and ZnO demonstrates the importance of morphology and macroscopic structural design for improving site accessibility and adsorbent recovery. These findings further confirm that uranium sequestration by metal oxides is governed not merely by oxide composition or specific surface area, but by the chemical identity, coordination environment, accessibility, and dynamic evolution of surface-active sites.

### 4.7. Mixed-Metal Oxides

Mixed-metal oxides have emerged as a versatile class of uranium sequestration because the incorporation of multiple metal centers provides greater flexibility in regulating surface chemistry, local coordination environments, defect structures, and interfacial properties than conventional single-metal oxides. Depending on their composition and architecture, different metal species can be incorporated into a common crystal lattice, distributed across heterogeneous oxide interfaces, or organized within layered structures. These structural characteristics generate chemically diverse metal-oxygen sites and provide opportunities to regulate surface hydroxylation and defect chemistry. In addition, the incorporation of magnetic components can facilitate adsorbent recovery. Therefore, the advantage of mixed-metal oxides lies not simply in combining the properties of individual oxides, but in creating new coordination environments and reactive interfaces for U(VI) sequestration.

Spinel ferrites are among the most representative mixed-metal oxides investigated for uranium adsorption. Hu et al. [79] synthesized magnetic MnFe2O4 nanocubes with an average particle size of approximately 50–70 nm and investigated their interaction with U(VI) using batch adsorption experiments, spectroscopic characterization, and density functional theory calculations. MnFe2O4 exhibited a maximum U(VI) Langmuir adsorption capacity of 119.90 mg·g^−1^ at pH 5.0 and 298.15 K, while its magnetic properties enabled convenient solid–liquid separation. The adsorption process was largely independent of ionic strength, indicating that uranium retention was dominated by specific inner-sphere interactions rather than nonspecific electrostatic association. FTIR and XPS analyses combined with theoretical calculations further indicated that metal-oxygen and surface hydroxyl groups were directly involved in U(VI) binding through surface complexation. The MnFe2O4 system therefore illustrates an important advantage of spinel mixed oxides, different metal-oxygen environments can be integrated within a stable crystalline framework while retaining magnetic recoverability.

Further improvement can be achieved by constructing heterogeneous interfaces between different oxide phases. Ding et al. [80] prepared TiO2@Zn Fe2O4 through an in situ fabrication strategy and obtained a porous mixed-oxide structure with substantially enhanced uranium adsorption. The optimized material exhibited a maximum Langmuir U(VI) adsorption capacity of 716.2 mg·g^−1^ and a removal efficiency of 98.3%, while more than 90% removal efficiency was retained after five adsorption–desorption cycles. The material also exhibited favorable selectivity toward U(VI) in the presence of competing ions. Mechanistic characterization suggested that uranium removal involved electrostatic attraction, surface complexation, and interactions associated with oxygen-vacancy sites. These results demonstrate that coupling two oxide phases can modify the local defect and coordination environment, generating adsorption sites that are not adequately described by the properties of either individual oxide alone.

The compositional complexity of mixed-metal oxides can be extended further through the incorporation of three different metal species. Yu et al. [81]. synthesized sphere-like Co-Mo-Ni ternary hydroxide (CMN) and subsequently converted it into a calcined ternary oxide material (CCMN). The maximum Langmuir U(VI) adsorption capacities of CMN and CCMN reached 585.6 and 961.5 mg·g^−1^, respectively, at pH 5.0 and 298 K, demonstrating that thermal conversion substantially altered the uranium adsorption performance of the multimetallic system. U(VI) uptake showed little dependence on ionic strength, while XPS analysis confirmed strong interactions between uranium species and metal–oxygen functional groups. The adsorption mechanism was therefore primarily attributed to inner-sphere surface complexation. Compared with conventional binary oxides, this ternary system provides a broader distribution of metal-oxygen coordination environments and demonstrates that compositional regulation, together with post-synthetic thermal treatment, can effectively modify the chemical reactivity of mixed-oxide surfaces toward uranium.

Another distinctive strategy involves layered mixed-metal oxides derived from layered double hydroxides (LDHs). Tao et al. [82] prepared Mg-Al layered double hydroxides and their calcined layered double oxide (LDO) counterparts using different drying treatments. Freeze-dried MgAl-LDO exhibited a maximum Langmuir U(VI) adsorption capacity of 1099.93 mg·g^−1^, substantially exceeding that of materials obtained through conventional oven drying. The improved adsorption was associated with its larger specific surface area and pore size as well as greater exposure of vacant sites within the layered structure. The relatively weak effect of ionic strength and the participation of M-O and C-O groups further indicated that chemical complexation played an important role in uranium retention. Unlike conventional spinel oxides, calcined layered double oxides possess a structurally responsive framework and can undergo surface and structural reconstruction in aqueous environments. Such characteristics provide an additional route for regulating uranium uptake through the combined effects of accessible coordination sites and dynamic structural evolution.

Taken together, these studies demonstrate a progressive evolution in the design of mixed-metal oxide adsorbents. Conventional spinel ferrites integrate multiple metal-oxygen sites with magnetic separability, whereas heterostructured oxides exploit interfacial and defect engineering to generate additional uranium-binding environments. Increasing compositional complexity through ternary oxides further expands the diversity of local coordination sites, while layered mixed-metal oxides introduce structurally responsive surfaces capable of reconstruction under aqueous conditions. The enhanced uranium sequestration performance of mixed-metal oxides should therefore not be interpreted simply as an additive effect of their constituent metals. Instead, their performance is governed by the interplay among metal composition, local M-O coordination, surface hydroxylation, defect chemistry, heterogeneous interfaces, and structural evolution. These characteristics make mixed-metal oxides particularly promising platforms for the rational design of next-generation uranium adsorbents. Beyond the rational design of high-performance uranium adsorbents, practical application also requires careful consideration of their regeneration capability, uranium recovery efficiency, structural stability during repeated adsorption–desorption cycles, and potential environmental risks associated with metal leaching. As summarized in Table 1, several representative metal oxide-based adsorbents exhibit promising recyclability, while detailed information regarding capacity retention, regeneration conditions, uranium recovery, structural evolution after regeneration, and the release of constituent metal species remains insufficient in many reported systems. For transition-metal and mixed-metal oxides, the possible dissolution of metal components such as Fe, Mn, Zn, Co, or Ni should be considered, particularly under acidic or chemically aggressive conditions, because metal release may affect their long-term environmental applicability. Therefore, future studies should combine adsorption–desorption evaluation with quantitative uranium recovery analysis, leaching assessment, and systematic characterization of regenerated adsorbents to better understand their practical feasibility.

## 5. Conclusions

Metal oxides provide versatile platforms for U(VI) sequestration because their adsorption sequestration can be regulated through both surface chemistry and structural design. Across the oxide systems reviewed here, uranium retention is primarily governed by interactions with surface hydroxyl and metal–oxygen sites, while surface precipitation and redox-assisted immobilization may additionally occur depending on oxide composition and aqueous conditions. Studies of Fe-, Ti-, Al-, Mn-, and other metal oxides demonstrate that crystal structure, morphology, surface functionalization, hydroxylation, and defects can substantially alter the availability and reactivity of uranium-binding sites, whereas mixed-metal oxides further expand these possibilities by introducing diverse coordination environments and heterogeneous interfaces. Overall, the performance of metal oxide adsorbents is determined not simply by their composition or surface area, but by the nature, accessibility, and structural regulation of active surface sites, which provides the fundamental basis for understanding and optimizing U(VI) sequestration by metal oxide-based materials.

## 6. Limitations and Perspectives

Despite significant progress in metal oxide-based uranium adsorbents, several challenges remain for their practical application. Future research should focus on the following aspects:

### 6.1. Validation Under Realistic Uranium-Containing Environments

Most studies are conducted using simplified laboratory solutions. Future investigations should evaluate adsorbent performance under realistic uranium-containing environments, including seawater, uranium-containing leachates, and complex aqueous matrices with competing ions or natural organic matter, to verify their applicability beyond idealized adsorption systems.

### 6.2. Mechanism-Guided Design of Next-Generation U(VI) Adsorbents

Future development should shift from empirical material screening toward mechanism-guided design by integrating oxide composition, surface structure, U(VI) speciation, and interfacial interactions. Surface hydroxyl groups, metal–oxygen coordination, defects, and heterogeneous interfaces should be rationally regulated based on experimentally verified uranium-binding mechanisms.

### 6.3. Regeneration, Uranium Recovery, and Long-Term Stability

Beyond adsorption capacity, future studies should evaluate regeneration efficiency, uranium recovery, structural stability, and potential metal leaching to establish the long-term applicability and environmental safety of metal oxide-based adsorbents.

### 6.4. Exploration of Advanced Oxide Architectures

Emerging oxide materials, such as high-entropy oxides and other compositionally complex systems, may provide new opportunities for uranium capture through tunable coordination environments and interfaces. However, their uranium retention mechanisms, stability, and practical advantages require further investigation.

## Figures and Tables

**Table 1:** Physicochemical characteristics, experimental conditions, and U(VI) removal and regeneration performance of representative metal oxide-based materials.

Material	Morphology and SSA (m^2^ g^−1^)	U(VI) Concentration	pH/Temperature (K)	Contact Time/Adsorbent Dosage	q_exp_/q_max_ (mg g^−1^)	Removal Efficiency	Regeneration/Reusability
IONPs@MAH-β-CD [49]	Mesoporous magnetic nanospheres, ~10–20 nm; 126.62	q_exp_: 40 mg L^−1^; q_max_: variable-concentration isotherm experiment	q_exp_: 6.0/303; q_max_: 6.0/313	q_exp_: 180 min/0.4 g L^−1^; q_max_: not separately restated in the isotherm section	87.02/163.93 (L.)	~70%	0.01 M HCl; removal remained >60.9% after 5 cycles
TiO_2_/Ti nanowire arrays [64]	Vertically aligned TiO_2_ nanowire arrays grown on Ti foil; NR	variable-concentration isotherm experiment	5.0/323	60 min/1.25 g L^−1^	NR/354.5 (L.)	~80 (C_0_ = 8 mg L^−1^, pH= 6–9, 298 K)	0.1 M HCl, 60 min; capacity essentially unchanged over 3 cycles; ~92% cumulative removal after 9 sequential treatments
TiO_2_ aerogel [65]	Three-dimensional porous TiO_2_ aerogel; NR	q_exp_: 10 mg L^−1^ q_max_: variable-concentration isotherm experiment	q_exp_: 5.0/298; q_max_: 5.0/298	q_exp_: 180 min/0.1 g L^−1^; q_max_: NR/0.1 g L^−1^	NR/638.0 (L.)	97.1%	Removal efficiency >90% after 5 cycles
Porous Al_2_O_3_-Cl [69]	Interconnected porous Al_2_O_3_; 258.3	10 mg L^−1^	7.0/298	NR/0.1 g L^−1^	NR/805.72 (L.)	95.4%	Removal efficiency >90% after 5 cycles
α-MnO_2_ [74]	α-MnO_2_ crystalline phase; 169.961	0.2 mmol L^−1^	5.5/298	75 min/0.2 g L^−1^	NR/≈280 (L.)	NR	NR
MXene/MnO_2_ [75]	MnO_2_ particles on layered MXene nanosheets; NR	120 mg L^−1^	7.03/298	4 min/5 mg	696/NR	nearly 100% (C_0_ = 120 mg L^−1^, pH ≈ 7)	NR
ZrO_2_/CS [76]	ZrO_2_/chitosan composite; 25.52	q_exp_: 200 mg L^−1^	pH 3.5/298	q_exp_:50 min/1 g L^−1^	175.0/175.44 (L.)	NR	0.8 M H_2_SO_4_, 75 min; desorption efficiency decreased from 98% to 82% after 7 cycles; U(VI) was effectively desorbed
Ce–OH-rich CeO_2_ [12]	Hollow CeO_2_ spheres enriched with surface Ce–OH groups; NR	0.1 mg L^−1^ and 1 mg L^−1^	4.6/298	≤1 min/0.2g L^−1^	NR/143.1	100%	NR
MgO-derived Mg(OH)_2_ [77]	Mesoporous Mg(OH)_2_ formed by hydrolysis of hierarchical porous MgO; 140	500 mg L^−1^	6/298	240 min/0.008 g	3111/NR	99%	1.0 M Na_2_CO_3_; U(VI) desorption yield reached 90.7% in the first cycle and remained 76.2% after three cycles
ZnO/CC [78]	ZnO nanorod arrays grown on cotton cloth, ~150–200 nm in diameter; NR	100–350 mg L^−1^	5.0/298	30 min/0.5 g L^−1^	NR/431.03 (L.)	NR	reconstructed ZnO/CC retained good adsorption ability after 3 cycles;
MnFe_2_O_4_ nanocubes [79]	Magnetic MnFe_2_O_4_ nanocubes, cube-shaped, ~50–70 nm; NR	10 mg L^−1^	5.0/298.15	4 h/0.2 g L^−1^	NR/119.90 (L.)	NR	0.1 M NaOH; no significant decrease in U(VI) removal efficiency after 5 cycles
TiO_2_@ZnFe_2_O_4_ [80]	Uniform porous structure; NR	10 mg L^−1^	4/298	2 h/0.1 g L ^−1^	NR/716.2 (L.)	98.3%	HCl solution (pH 2); removal remained >90% after 5 cycles

Note: SSA, specific surface area; q_exp_, experimentally measured adsorption capacity; q_max_, model-derived maximum adsorption capacity; L., Langmuir model; NR, not reported in the original reference.

## Data Availability

No new data were created or analyzed in this study. Data sharing is not applicable to this article.
